# Assessment of Cone-Beam Breast Computed Tomography for Predicting Pathologic Response to Neoadjuvant Chemotherapy in Breast Cancer: A Prospective Study

**DOI:** 10.1155/2022/9321763

**Published:** 2022-04-29

**Authors:** Shen Chen, Sheng Li, Chunyan Zhou, Ni He, Jieting Chen, Shengting Pei, Jiao Li, Yaopan Wu, Peiqiang Cai

**Affiliations:** ^1^Department of Medical Imaging and Image-Guided Therapy, Sun Yat-Sen University Cancer Center, State Key Laboratory of Oncology in South China, Collaborative Innovation Center for Cancer Medicine, Dongfeng Dong Road, Guangzhou 510060, China; ^2^Guangdong Provincial Key Laboratory of Biomedical Imaging and Guangdong Provincial Engineering Research Center of Molecular Imaging, The Fifth Affiliated Hospital, Sun Yat-Sen University, Zhuhai, Guangdong Province 519000, China

## Abstract

**Background:**

Response surveillance of neoadjuvant chemotherapy is needed to facilitate treatment decisions. We aimed to assess the imaging features of cone-beam breast computed tomography (CBBCT) for predicting the pathologic response of breast cancer after neoadjuvant chemotherapy.

**Methods:**

This prospective study included 81 women with locally advanced breast cancer who underwent neoadjuvant chemotherapy from August 2017 to January 2021. All patients underwent CBBCT before treatment, and 55 and 65 patients underwent CT examinations during the midtreatment (3 cycles) and late-treatment phases (7 cycles), respectively. Clinical information and quantitative parameters such as the diameter, volume, surface area, and CT density were compared between pathologic responders and nonresponders using the *T*–test and the Mann–Whitney *U* test. The performance of meaningful parameters was evaluated with the receiver operating characteristic curve, sensitivity, and specificity.

**Results:**

The quantitative results for the segmented volume, segmented surface area, segmented volume reduction, maximum enhancement ratio, wash-in rate and two-minute enhancement value in the mid- and late-treatment periods had predictive value for pathologic complete response. The area under the curve for the prediction model after multivariate regression analysis was 0.874.

**Conclusion:**

After comparing the outcomes of each timepoint, mid- and late-treatment parameters can be used to predict pathologic outcome. The late-treatment parameters showed significant value with a predictive model.

## 1. Introduction

Neoadjuvant chemotherapy (NAC) is the standard systemic treatment for locally advanced breast cancer before surgery and radiation therapy [1]. It reduces tumor staging, facilitates the maneuverability of inoperable masses, and elevates the quality of breast-conserving surgery (BCS) [2]. Tumor response during and after treatment is associated with long-term prognosis [3]. Pathologic complete response (pCR) generally indicates longer disease-free survival and overall survival compared with cases involving residual tumor [4]. Furthermore, individual susceptibility to NAC varies with genetic variation [5, 6]. Therefore, it is sensible to help clinicians make a precise evaluation of therapeutic efficacies and further fine-tune individualized prescriptions.

The application of cone-beam breast computerized tomography (CBBCT) in diagnosing breast diseases is relatively new [7]. This technique exerts no pressure on the glands on examination, avoiding possible tumor spread due to compression [8]. The radiation dose is comparable to that of mammography and performs well for dense, glandular lesions [9]. It has comparable sensitivity in identifying benign and malignant lesions to magnetic resonance imaging [10, 11]. Previous studies have shown that CBBCT is helpful in distinguishing tumor subtypes [12–14]. A recent study found a novel approach to lesion classification by developing a convolutional neural network with deep learning [15]. It has also been shown to demonstrate diagnostic value for nonmass enhancement during breast imaging [16]. Calcification is an early manifestation of intraductal breast cancer, and reduced or increased calcification during chemotherapy may represent the metabolic reaction of tumor cells during treatment. CBBCT has a unique advantage in the stereoscopic display of calcification [17] and can monitor the calcification component of tumors during neoadjuvant chemotherapy.

Quantitative parameters for predicting the response to neoadjuvant chemotherapy have been reported using different imaging modalities. Although prediction performance varies among different imaging predictors, establishing optimal timepoints before and during NAC has been considered a concern in some studies [18]. In addition, some early studies primarily applied two-dimensional measurements of lesions rather than volumetric parameters to predict pathologic response [19]. By comparison, the innovation of this study is the three-dimensional measurement and hemodynamic analysis of lesions during chemotherapy. This study was aimed at analyzing the parameters of CBBCT and exploring its predictive value for pathologic response during different periods of neoadjuvant chemotherapy.

## 2. Methods

### 2.1. Study Eligibility and Population

This prospective study was conducted in compliance with the Health Insurance Portability and Accountability Act. The Sun Yat-sen University Affiliated Cancer Center institutional review board approved the study, and all patients were provided informed consent before their hospitalization.

**Figure 1:**
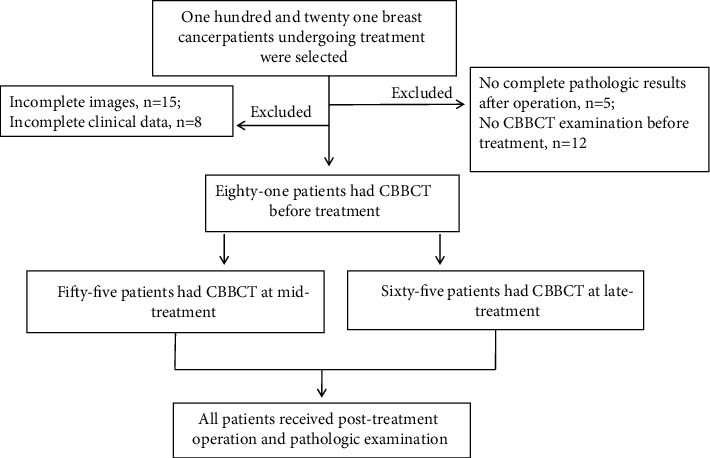
Population study flowchart depicting CBBCT examination at each timepoint. CBBCT: Cone-beam breast computed tomography.

**Figure 2:**
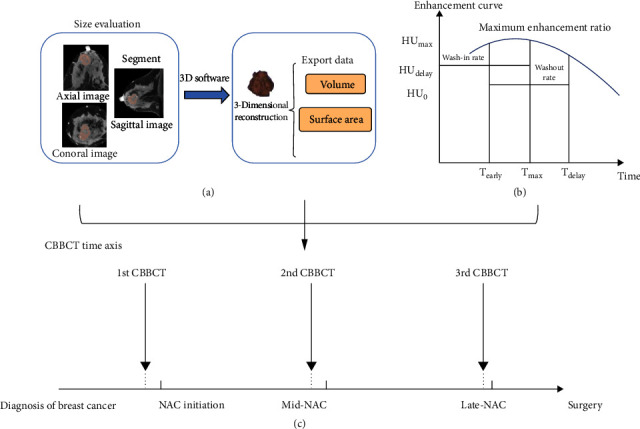
A graphical description of the procedure measuring size (a), illustration of enhancing curves (b), and CBBCT examination timepoints during chemotherapy (c). NAC: Neoadjuvant chemotherapy; CBBCT: Cone-beam breast computed tomography.

A total of 121 women with needle biopsy-proven locally advanced breast cancer scheduled for NAC participated in this study between August 2017 and January 2021. All patients received 4−8 cycles of doxorubicin or paclitaxel-based NAC with/without targeted therapy, followed by surgery and pathologic assessment of surgical specimens. Among them, 81 patients received routine CBBCT examination before therapy, 55 patients received examination at the midtreatment phase (3 cycles), and 65 patients received CT examinations at the late-treatment phase (7 cycles). A flow chart of the study design, including the patient population and CT examination timepoints, is illustrated in Figures 1 and 2(c). The study's exclusion criteria were as follows: (A) no pathologic assessment after NAC; (B) only one CBBCT examination performed before NAC; (C) only plain images available or the lack of some enhancement stages; and (D) incomplete baseline information.  Table 1 summarizes the baseline clinicopathologic features.

### 2.2. CBBCT Protocol

CBBCT examinations were performed at our institution using cone-beam breast CT (KBCT1000, Koning Corporation). The examinations for patients were conducted in a prone position. All patients underwent CBBCT examinations, including during the no angiography period and during angiography at 60 s, 120 s, and 180 s. Iodine contrast agent was intravenously injected using a high-pressure injector at a 1-2 ml/kg concentration and speed of 2.0-3.0 ml/s. In this study, the X-ray tube voltage was constant at 49 kVp, while the tube current was 50-160 mA (calculated automatically based on the size and density of the breast examined). Three-dimensional (3D) stereoscopic reconstruction with a voxel size of 0.273 mm^3^ was performed using a soft tissue filter in the standard mode.

### 2.3. Image Evaluation

Two radiologists specializing in breast imaging diagnosis independently interpreted the CBBCT images before reading relevant clinical information for each cycle of NAC. A consensus was reached in case of discrepancy. Quantitative measurements were performed on cancerous foci. For patients with multiple lesions, we measured the largest lesion.

Multiplanar reconstruction on noncontrast-enhanced (CE) and CE-CBBCT with a slice thickness of 2.7 mm was performed by three-dimensional visualization software and a postprocessing workstation (Visage CS Thin Client) for this purpose. Parameters (including diameter and lesion enhancement (*Δ*HU)) were evaluated at the workstation using a specific measurement tool. Diameters were calculated as the maximum length of the tumor's largest cross-section. Tumor volume was calculated with the ellipsoidal formula as described in reference (20). Reduction of the tumor at each timepoint of NAC was calculated with the baseline measurement of mass volume before chemotherapy. To evaluate the Hounsfield units (HUs) within lesions, a region of interest was placed on the largest section of the lesion while avoiding observable necrosis. Lesion enhancement at each phase was recorded according to the following formula [21]: ΔHU = (HU_lesion−post_ − HU_fat−post_) − (HU_lesion−pre_ − HU_fat−pre_). Thereafter, tissue density measurements within the same layer were standardized.

The three-stage enhancement characteristics were the maximum enhancement ratio, wash-in rate, and washout rate. An example enhancement curve is shown in Figure 2(b).

- The maximum enhancement ratio is the peak enhancement value minus the CT value before enhancement divided by the CT value before enhancement: maximum enhancement ratio% = (ΔHU_max_ − ΔHU_0_)/ΔHU_0_
- The wash-in rate is the absolute value of enhancement divided by the scanning time of the phase: wash − in rate%/*s* = (ΔHU_max_ − ΔHU_0_)/*T*_max_
- The washout rate is defined as the difference in the enhancement value in the delayed phase and the earlier enhancement value divided by the time between the two phases: washout rate%/*s* = (ΔHU_early_ − ΔHU_delay_)/*T*_delay_ − *T*_early_

Two radiologists segmented the lesions using a 3D visualization tool (3D slicer) segmentation module on the CE-CBBCT scans. The dedicated software uses a threshold method to determine the range of editable threshold values, automatically segmenting the enhanced tumor. The segmented image was displayed as multiplanar reconstruction images and converted to 3D form (as shown in Figure 2(a)). The volume and surface area were calculated automatically by the software. Finally, all derived features were subsequently reviewed by a senior radiologist (with at least ten years of breast imaging experience).

### 2.4. Histopathologic Evaluation

Patients were dichotomized into pCR and non-pCR groups according to surgical anatomical specimens. The pathologic response of the surgical specimens was assessed by the Miller-Payne grading (MPG) system, which compares the pathology before treatment with that of surgical specimens obtained after treatment to assign one of five Grades according to the richness of the residual tumor cells [22, 23]: Grade 1, no reduction in overall cellularity; Grade 2, a minor loss of tumor cells (up to 30% loss); Grade 3, an estimated decrease in tumor cells from 30% to 90%; Grade 4, significant loss of tumor cells (more than 90%); and Grade 5, no identifiable malignant cells, although ductal carcinoma in situ may be present. Grade 5 was defined as pCR, and Grades 1-4 were defined as non-pCR. Histologic subtypes were classified into luminal A or B type, (estrogen receptor (ER) or progesterone receptor (PR) positive); human epidermal growth factor receptor 2- (HER2-) enriched type (ER and PR negative and HER2-positive); and triple-negative type (ER, PR, and HER2-negative). According to the level of Ki67 receptor, the samples were divided into high and low proliferation (Ki67>14% vs ≤14%, respectively).

### 2.5. Statistical Analysis

Analysis of patient baseline data and imaging parameters ass assessed by CBBCT was performed with the statistical software SPSS 26 (IBM, Armonk, NY, USA). The chi-square test or Fisher's exact test was used for categorical variables of the pCR and non-pCR groups. All statistical hypothesis tests were two-sided. *p* values less than 0.05 were considered significant in all analyses. *T*–tests and Mann–Whitney *U* tests were used for continuous data. Univariate and multivariate logistic regression with the backward stepwise method were applied to select the key factors for predicting pCR. The area under the receiver operator characteristic curve (AUC), cutoff, sensitivity, and specificity was calculated in the diagnostic test.

## 3. Results

### 3.1. Clinical and Baseline Characteristics

A total of 81 female patients with breast cancer were assessed. According to the final pathologic results, 36 patients (44.4%) were placed in the pCR group, and 45 (55.6%) were placed in the non-PCR group. Among the clinical features summarized in Table 1, age significantly differed between the pCR group and the non-pCR group (*p* = 0.003). The mean age was 43.4 years (ranging from 26 to 67 years). The median diameter of the tumor was 41.3 mm (ranging from 10 to 97 mm), and with 20 and 50 mm as classifications of the tumor diameter, a significant difference was observed between the two groups (*p* = 0.043). There was no significant difference in other clinicopathologic features (*P* > 0.05).

### 3.2. Quantitative Parameters of CBBCT before and during NAC

**Figure 3:**
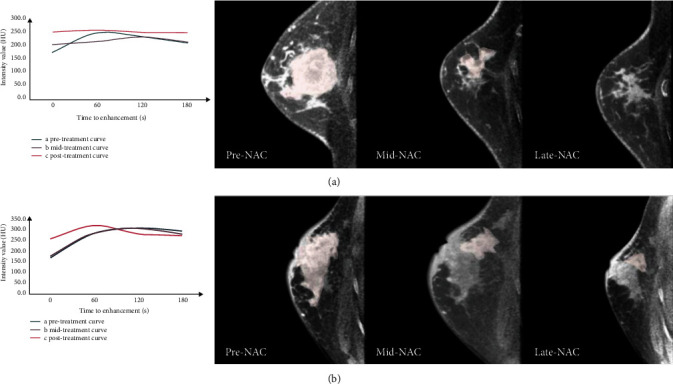
Sagittal images before and during neoadjuvant chemotherapy of pathologic responder (a) and nonresponder (b) from 56-year-old and 31-year-old women with right side invasive breast cancer, respectively. The enhancement curve was plotted by measuring density changes in each mass during the enhancement scan. NAC: Neoadjuvant chemotherapy.

Table 2 summarizes the quantitative parameters of the pCR and non-PCR patients pre-NAC and during NAC. Before treatment, the parameters were not statistically different (*p* > 0.05). At the midtreatment phase, most enhancement parameters (including two-minute and three-minute enhancement, wash-in rate, and maximum enhancement ratio) were statistically significant. The magnitude and variation of lesion diameter, volume, and surface area were also significantly different between the pCR and non-PCR groups. Furthermore, most parameters measured at the late stage of treatment were significantly different (*p* value ranging from 0.001 to 0.017), except for the washout rate.  Figure 3 describes tumor change before and during neoadjuvant chemotherapy. Each stage of chemotherapy includes an enhancement curve detailing tumors' features.

### 3.3. Predictive Value for pCR at the Midtreatment Phase

Fifty-five (68.0%) patients were examined at the midtreatment phase. Among size-related predictors, diameter, volume, segmented volume, and segmented surface area were statistically significant (*p* < 0.05) (Supplementary tables). Among enhancement-related predictors, two-minute enhancement, wash-in rate, and maximum enhancement ratio had predictive value in our assessment. The median segmented tumor volume was 1906.5 mm^3^ (ranging from 86.1 mm^3^ to 90,290.6 mm^3^), which was significantly correlated with pCR (AUC = 0.729, *p* = 0.004), and the cutoff segmented volume reduction was 91.5% (AUC = 0.677, *p* = 0.026). At this timepoint, the highest sensitivity (76.7%) and specificity (70.8%) were achieved for segmented volume using a cutoff of 1520.4 mm^3^. After two minutes of enhancement, the pCR prediction value was the highest among the parameters at the midtreatment phase, with an AUC value of 0.759 (*p* = 0.001). All calculated AUCs were compared in pairs using Delong's test.

### 3.4. Predictive Value for pCR at the Late-Treatment Phase

**Figure 4:**
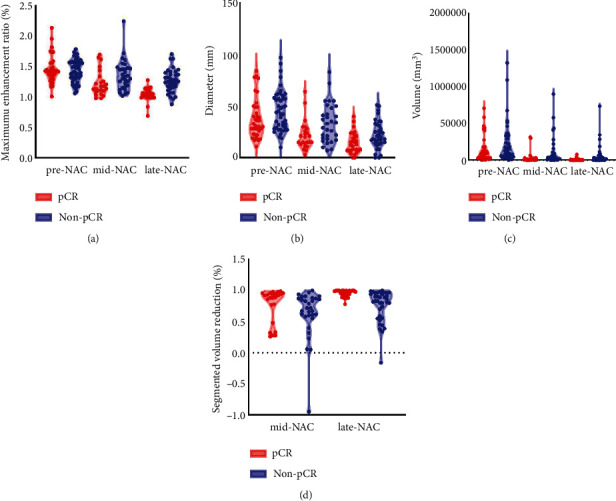
Parameters of maximum enhancement ratio (a), diameter (b), volume (c), and segmented volume reduction (d) for pCR and non-pCR groups at each timepoints of NAC. pCR: Pathologic complete response; NAC: Neoadjuvant chemotherapy.

**Figure 5:**
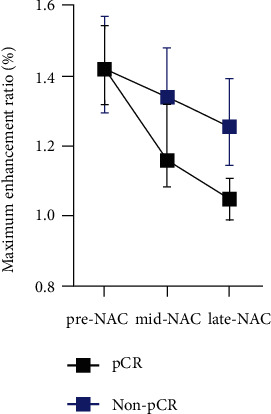
Maximum enhancement ratio of pCR and non-pCR groups at three examination's time before and during neoadjuvant therapy. pCR: Pathologic complete response; NAC: Neoadjuvant chemotherapy.

Sixty-five (80.2%) patients examined by CBBCT were evaluated at the late-treatment phase. The predictive values of most parameters in the late treatment were higher than those in the middle treatment (AUC = 0.689 ~ 0.837). Except for the washout rate, most parameters at the late-treatment phase significantly predicted pathologic status (*p* < 0.05). The responders' size and enhancement were significantly lower (*p* = 0.001 ~ 0.037) than those of the group of nonresponders before and during NAC (Figure 4). The AUC of the maximum enhancement ratio was 0.837 (0.735, 0.938), outperforming the other parameters. It had a high sensitivity of 96.6%, and the cutoff value was 1.25% (Supplementary tables). It should be noted that this parameter changed during chemotherapy. As Figure 5 demonstrates, the variation between pathologic responders and nonresponders became gradually distinct as they approached the end of therapy. Additionally, the segmented volume and segmented surface area (AUC = 0.791 and 0.766, *p* < 0.001) showed high predictive value. For parameters at each timepoint, the AUC values were compared with the Delong test to prove the higher diagnostic performance within each parameters.

### 3.5. A Predictive Model for pCR based on Univariable and Multivariable Analysis

**Figure 6:**
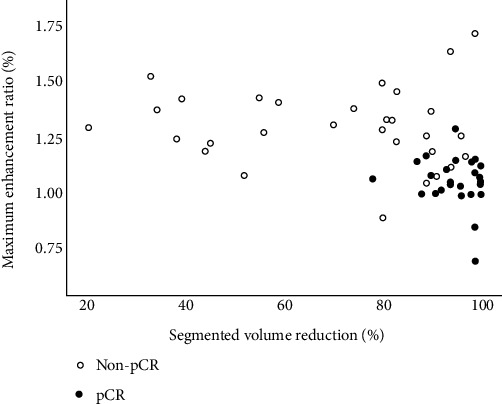
Correlation between segmented volume reduction and maximum enhancement ratio of pCR and non-pCR groups at late-treatment. pCR: Pathologic complete response.

We constructed a predictive model based on univariable and multivariable regression analysis parameters. Univariable analysis for the midtreatment parameters showed that four were statistically significant, namely, two-minute enhancement, maximum enhancement ratio, diameter, and hormone status (*p* < 0.05). Multivariable analysis showed no significant results (*p* > 0.05) (Supplementary tables). At the late-treatment phase, multivariable regression showed that the maximum enhancement ratio had an odds ratio (OR) of 2.192 and a *p* value of 0.002 as a risk predictor of pathologic status, while a reduction in segmented volume (OR = 0.319, *p* = 0.02) was a protective predictor (Table 3). Subsequently, a predictive model for the late-treatment phase was constructed with the selected parameters.  Figure 6 depicts the distribution of these two parameters between responders and nonresponders at the late-treatment phase. For pCR, as masses start to shrink more with increased chemotherapy time, the increasing volume reduction rate is further correlated with pathologic status. Finally, the performance of the predictive model was evaluated. At a cutoff of 0.278, the model showed a predictive value of 0.874, a sensitivity of 0.966, and a specificity of 0.694.

## 4. Discussion

Neoadjuvant chemotherapy is crucial in the treatment of advanced breast cancer patients, reducing the preoperative tumor volume, downgrading the tumor stage, and improving the long-term prognosis. The subject of this study is the prediction of tumor pathologic response by CBBCT in the setting of NAC. Our study reveals valuable CBBCT parameters for predicting pCR (*p* < 0.05) at the middle and late phases of NAC. Furthermore, the AUC of the prediction model at the late treatment phase was 0.874, suggesting that CBBCT can provide valuable information for the pathologic outcome of NAC.

Tumor diameter was measured according to the Response Evaluation Criteria in Solid Tumors for evaluating tumor efficacy [24]. In concordance with the study of Fangberget et al., the volume measurement outperformed the unidimensional measurement during NAC [25]. This study investigated the predictive value of surface area on NAC response for the first time using 3D imaging software. We found that volume measurements had more predictive value than diameter and surface area in terms of the AUCs. Moreover, the segmented volume measurements demonstrated better predictive value than the calculated size, indicating the potential of a flexible automated NAC evaluation method.

The evaluation of volume and volumetric reduction by various imaging modalities (e.g., mammography, ultrasound) for monitoring pathologic response was studied previously [22, 26]. Although volume measurements in NAC may vary depending on the image evaluation criteria, many articles have consistently supported the predictive value of volume measurements for evaluating the pathologic response. A feasibility study by Vedantham et al. demonstrated that evaluating tumor size helped monitor the response. It was found that tumor volume and volume changes on noncontrast-enhanced CBBCT during NAC may contribute to predicting pathologic outcomes [23]. Our study identified the predictive value of volume during late-phase NAC, which may be due to the major reduction in tumor size during this treatment period. The cutoff value for size reduction over 7 cycles was 86%, with a sensitivity of 61% and a specificity of 86%, which is consistent with earlier literature [25]. As masses gradually shrink over the duration of chemotherapy, the increasing volume reduction may further correlate with pathologic status.

CBBCT enhancement parameters showed predictive efficacy in predicting response. Imaging parameters capable of revealing the vascular kinetics of tumors and enhanced areas are important for monitoring the effects of chemotherapy and predicting tumor prognosis. Previous studies have revealed the predictive value of dynamic-enhanced CT and magnetic resonance imaging (MRI) in predicting pCR [27]. Functional parameters, for instance, the apparent diffusion coefficient in diffusion weighted imaging, as suggested in the literature, can provide accurate internal information prior to the observation of size change [28]. Dynamic tissue perfusion changes, and enhancement in the internal and surrounding glandular areas of invasive tumors may suggest curative outcomes [29]. Similar results were found in our study: such dynamic enhancement inside the tumor predicted pathologic status, as indicated by the predictive values of the wash-in rate and maximum enhancement ratio at the midtreatment and late-treatment phases. Ohashi et al. presented a predictive MRI model generated from logical regression analyses and suggested that maximum slope may be valuable in evaluating the pathologic response [30]. Although the washout rate in this study had no predictive value, some studies have found significant results with textual analysis of tumor washout [31].

The predictive value of contrast enhancement at the two-minute phase may suggest an ideal time for evaluation. A similar study by Uhlig et al. [32] showed that CE-CBBCT images obtained two minutes after intravenous administration of contrast agent facilitated the differentiation of malignant and benign breast lesions.

As pCR is correlated with long-term prognosis after NAC and surgery, monitoring the tumor response is important for evaluating drug effectiveness [33]. HER2-positive patients would benefit substantially from NAC, although accuracy may be influenced by trastuzumab therapy [34]. Our study found that the HER2-positive population achieved more pathologic responders than the HER2-negative population, suggesting that HER2 overexpression is a positive predictive factor. However, this difference was not statistically significant in our analysis. Univariable regression analysis showed that hormone receptor status was a strong predictor for pCR outcome. Previous studies have reported the effect of hormone expression on favorable pCR outcomes [35]. These findings suggest that identifying tumor responders to NAC would be convenient for potential pCR patients.

In studies of dynamic contrast-enhanced- (DCE-) MRI combinations of multiple features achieved a higher AUC than individual features [36, 37]. Imaging biomarkers that monitor tumor volume based on pharmacokinetic thresholds demonstrated predictive value for pCR [37]. Our CBBCT model predicted the chemotherapy response with an accuracy of 81.5 and a sensitivity of 99.6%, which is consistent with many studies [33]. However, the cumulative false-positive rate reduces the specificity of CBBCT, while MRI parameters (such as the apparent diffusion coefficient, which was higher in responders) can be more effective in distinguishing negativity, as reported in the literature [38]. In fact, near-pCR patients generally demonstrated fewer residual tumors and low enhancement, which may be concealed by enhancing inflammation and fibrosis after NAC [39]. These factors will affect judgment. Therefore, in the middle and later stages of chemotherapy, CBBCT has high accuracy for predicting positive lesions, but the low accuracy of judging negative lesions may be one of its limitations.

Our study had several limitations. First, the patient population of this prospective study at each timepoint was small. Future studies with a larger population are warranted to validate our results. Second, the number of patients required to perform subgroup analysis by therapy regimen was inadequate, and selection bias may occur. Third, an additional independent dataset should be used to test the generalizability of our constructed model at the late-treatment phase.

## 5. Conclusion

CBBCT examination in the course of NAC therapy can assist in the evaluation of chemotherapy efficacy and predict pathologic outcomes. We hope that the predictive performance of our response assessment model will be beneficial clinical decision-making.

## Figures and Tables

**Table 1:** Baseline characteristics of patients.

Characteristics	pCR (*n* = 36)	Non-pCR (*n* = 45)	*p* value
*Side*			
Left	15 (41.7)	19 (42.2)	0.594
Right	21 (58.3)	26 (57.8)	
Age (years)	43.36 ± 8.49	44 ± 10	0.003^∗^
*Body mass index*			0.806
Low	2 (5.6)	3 (0.6)	
Normal	24 (66.7)	30 (66.7)	
Obesity	8 (22.2)	10 (22.2)	
Overweight	2 (5.6)	2 (0.5)	
*Menopausal status*			0.447
Premenopausal	30 (83.3)	37 (82.2)	
Postmenopausal	6 (16.7)	8 (17.8)	
*Family history*			0.786
With	7 (19.4)	9 (20.0)	
Without	29 (80.6)	36 (80.0)	
*Therapy*			0.078
Doxorubicin	0 (0)	0 (10.3)	
Paclitaxel without trastuzumab target	22 (61.1)	27 (51.7)	
Paclitaxel with trastuzumab target	14 (38.9)	18 (37.9)	
Tumor baseline size (mm)	32.4 (24.5, 48.1)	44.6 (29.2, 58.1)	0.067
*Histologic type*			0.246
Ductal carcinoma in situ	1 (2.8)	1 (2.2)	
Invasive ductal carcinoma	34 (94.4)	43 (95.6)	
Mixing invasive carcinoma	1 (2.8)	1 (2.2)	
*Hormone receptor*			0.981
Positive negative	15 (41.7)	1832 (40.071.1)	
Negative positive	21 (58.3)	2713 (6028.9.0)	
*HER2*			0.951
Negative	6 (16.7)	7 (15.6)	
Positive	30 (83.3)	38 (84.4)	
*Ki-67 receptor*			0.197
Low	2 (5.6)	3 (6.7)	
High	34 (94.4)	42 (93.3)	
*Complications*			0.439
With	7 (19.4)	9 (20.0)	
Without	29 (80.6)	36 (80.0)	

Note: The data reveals numbers of patients in the pCR and non-pCR group with percentages in parentheses or mean ± standard deviation. *p* values marked asterisk present significant statistical value. pCR: Pathologic complete response; HER: Human epidermal growth factor receptor.

**Table 2:** CBBCT parameters before and during NAC.

Parameters	Pre-NAC	*p* value	Mid-NAC	*p* value	Late-NAC	*p* value
Lesion density/HU	204.7 ± 42.4	0.999	205.5 ± 47.3	0.671	203.6 ± 42.8	0.756
One-min enhancement/HU	75.8 ± 39.1	0.794	35.6 ± 58.1	0.156	16.3 ± 34.5	0.001∗
Two-min enhancement/HU	75.8 ± 35.6	0.898	47.6 ± 38.4	0.007∗	23.8 ± 44.6	0.001∗
Three-min enhancement/HU	73.5 ± 37.3	0.621	42.0 ± 49.5	0.046∗	22.3 ± 36.9	0.011∗
Diameter/mm	41.3 ± 19.1	0.064	27.1 ± 16.7	0.006∗	18.7 ± 13.1	0.012∗
Volume/mm^2^	12873.0 ± 34946.8	0.062	3521.3 ± 18795.6	0.002∗	1141.1 ± 9902.9	0.017∗
Segmented volume/mm^3^	9666.7 ± 20240.7	0.082	1906.5 ± 16845.8	0.004∗	914.5 ± 10699.8	<0.001∗
Segmented surface area/mm^2^	9487.6 ± 23594.4	0.072	3087.5 ± 19916.3	0.013∗	1542.8 ± 13681.1	<0.001∗
Washout rate (%/s)	0.09 ± 0.138	0.422	0.17 ± 1.182	0.056	0.06 ± 2.083	0.622
Maximum enhancement ratio (%)	1.42 ± 0.202	0.97	1.30 ± 0.241	0.023∗	1.17 ± 0.192	<0.001∗
Wash-in rate (%/s)	0.24 ± 0.186	0.921	0.173 ± 0.206	0.034∗	0.089 ± 0.123	<0.001∗

Note: The numbers in the table represent mean with standard deviation. *p* values with statistical value are marked with asterisk. HU: Hounsfield unit. CBBCT: Cone-beam breast computed tomography. NAC: Neoadjuvant chemotherapy.

**Table 3:** Univariable and multivariable regression analysis of CBBCT parameters in late-NAC.

	Univariable analysis of CBBCT parameters in late-NAC			Multivariable analysis of CBBCT parameters in late-NAC		
	OR	SE	*p* value	OR	SE	*p* value
(Reference)	1			1		
Hormone status	0.269 (0.096,0.755)	0.527	0.013			
One-min enhancement/HU	0.990 (0.980, 1.000)	0.005	0.048			
Two-min enhancement/HU	0.986 (0.976, 0.996)	0.005	0.007			
Three-min enhancement/HU	0.984 (0.972, 0.996)	0.006	0.008			
Wash-in rate (%/s)	4.293 (1.770, 10.413)	0.452	0.001			
Maximum enhancement ratio (%)	2.667 (1.620, 4.390)	0.254	<0.001	2.192 (1.338, 3.590)	0.252	0.002
Diameter/mm	0.946 (0.903, 0.991)	0.024	0.020			
Segmented volume/mm^3^	2.833 (1.633, 4.916)	0.281	<0.001			
Segmented surface area/mm^2^	1.967 (1.278, 3.028)	0.220	0.002			
Segmented volume reduction (%)	0.264 (0.111, 0.628)	0.441	0.003	0.319 (0.122, 0.836)	0.492	0.02
Segmented surface area reduction (%)	0.468 (0.286, 0.764)	0.25	0.002			

Note: The numbers in the table represent point estimate with 95% confidence intervals. *p* values with statistical value are marked with asterisk. HU: Hounsfield unit. OR: odds ratio. SE: Standard error. CBBCT: cone-beam breast computed tomography. NAC: Neoadjuvant chemotherapy.

## Data Availability

The datasets used and/or analyzed during the current study are available from the corresponding author on reasonable request.

## Abbreviations

CE-CBBCT: Contrast-enhanced cone-beam breast computerized tomography
DCE-MRI: Dynamic contrast-enhanced magnetic resonance imaging
NAC: Neoadjuvant chemotherapy
pCR: Pathologic complete response
HU: Hounsfield unit
AUC: The area under the receiver operator characteristic curve
OR: Odds ratio
ER: Estrogen receptor
PR: Progesterone receptor
HER: Human epidermal growth factor receptor.

## Supplementary Materials

Supplementary Materials are enclosed herewith. Please find “Supplementary file.docx.”
