# Supplementary material for: Assessment of Cone-Beam Breast Computed Tomography for Predicting Pathologic Response to Neoadjuvant Chemotherapy in Breast Cancer: A Prospective Study
Source: J Oncol. 2022 Apr 29;2022:9321763. doi: 10.1155/2022/9321763 (PMC9076291; doi:10.1155/2022/9321763)
Supplement: Supplementary Materials — are enclosed herewith. Please find “Supplementary file.docx.” [file 9321763.f1.docx]

| **Patients** | **Histological type** | **Ki-67** | **Molecular type** | **Her 2** | **ER/PR** | **MP grade** | **Enrollment** |
| --- | --- | --- | --- | --- | --- | --- | --- |
| 1 | Invasive ductal carcinoma | High | Her 2 | Positive | Negative | 5 | Include |
| 2 | Invasive ductal carcinoma | High | Her 2 | Positive | Negative | 5 | Include |
| 3 | Ductal carcinoma in situ | High | Luminal B | Positive | Positive | 4 | Include |
| 4 | Invasive ductal carcinoma | High | Luminal B | Positive | Positive | 4 | Include |
| 5 | Invasive ductal carcinoma | High | TNBC | Negative | Negative | 2 | Include |
| 6 | Invasive ductal carcinoma | High | Luminal B | Positive | Positive | 5 | Include |
| 7 | Invasive ductal carcinoma | Low | Luminal A | Positive | Positive | 3 | Include |
| 8 | Invasive ductal carcinoma | High | Luminal B | Positive | Positive | 4 | Include |
| 9 | Invasive ductal carcinoma | Low | Luminal A | Positive | Positive | 3 | Include |
| 10 | Invasive ductal carcinoma | High | Her 2 | Positive | Negative | 4 | Include |
| 11 | Invasive ductal carcinoma | High | Luminal B | Positive | Positive | 5 | Include |
| 12 | Invasive ductal carcinoma | High | Luminal B | Positive | Positive | 5 | Include |
| 13 | Invasive ductal carcinoma | High | Luminal B | Positive | Positive | 3 | Include |
| 14 | Invasive ductal carcinoma | Low | TNBC | Negative | Negative | 5 | Include |
| 15 | Invasive ductal carcinoma | High | Luminal B | Positive | Positive | 2 | Include |
| 16 | Invasive ductal carcinoma | High | Her 2 | Positive | Negative | 5 | Include |
| 17 | Invasive ductal carcinoma | High | Her 2 | Positive | Negative | 5 | Include |
| 18 | Invasive ductal carcinoma | High | Her 2 | Positive | Negative | 5 | Include |
| 19 | Invasive ductal carcinoma | High | Luminal B | Positive | Positive | 2 | Include |
| 20 | Invasive ductal carcinoma | High | Her 2 | Positive | Negative | 5 | Include |
| 21 | Invasive ductal carcinoma | High | Luminal B | Positive | Positive | 5 | Include |
| 22 | Invasive ductal carcinoma | Low | TNBC | Negative | Negative | 5 | Include |
| 23 | Invasive ductal carcinoma | High | Her 2 | Positive | Negative | 4 | Include |
| 24 | Invasive ductal carcinoma | High | Her 2 | Positive | Negative | 5 | Include |
| 25 | Invasive ductal carcinoma | High | Her 2 | Positive | Negative | 3 | Include |
| 26 | Invasive ductal carcinoma | High | Her 2 | Positive | Negative | 5 | Include |
| 27 | Invasive ductal carcinoma | High | Luminal B | Positive | Positive | 5 | Include |
| 28 | Mixing invasive carcinoma | High | Her 2 | Positive | Negative | 5 | Include |
| 29 | Invasive ductal carcinoma | High | Luminal B | Positive | Positive | 5 | Include |
| 30 | Invasive ductal carcinoma | High | Luminal B | Negative | Positive | 4 | Include |
| 31 | Invasive ductal carcinoma | High | Luminal B | Positive | Positive | 5 | Include |
| 32 | Invasive ductal carcinoma | High | Her 2 | Positive | Negative | 5 | Include |
| 33 | Invasive ductal carcinoma | High | Luminal B | Positive | Positive | 3 | Include |
| 34 | Invasive ductal carcinoma | High | Luminal A | Positive | Positive | 2 | Include |
| 35 | Invasive ductal carcinoma | High | Luminal B | Positive | Positive | 1 | Include |
| 36 | Invasive ductal carcinoma | High | Luminal B | Negative | Positive | 5 | Include |
| 37 | Invasive ductal carcinoma | High | Her 2 | Positive | Negative | 5 | Include |
| 38 | Invasive ductal carcinoma | High | Luminal B | Negative | Positive | 2 | Include |
| 39 | Invasive ductal carcinoma | High | Luminal B | Positive | Positive | 2 | Include |
| 40 | Invasive ductal carcinoma | High | Luminal B | Positive | Positive | 4 | Include |
| 41 | Invasive ductal carcinoma | High | Luminal B | Positive | Positive | 5 | Include |
| 42 | Invasive ductal carcinoma | High | Her 2 | Positive | Negative | 5 | Include |
| 43 | Invasive ductal carcinoma | High | Luminal B | Positive | Positive | 3 | Include |
| 44 | Invasive ductal carcinoma | High | Luminal B | Positive | Positive | 2 | Include |
| 45 | Invasive ductal carcinoma | High | Luminal B | Positive | Positive | 3 | Include |
| 46 | Invasive ductal carcinoma | High | Her 2 | Positive | Negative | 2 | Include |
| 47 | Invasive ductal carcinoma | High | Luminal B | Positive | Positive | 2 | Include |
| 48 | Invasive ductal carcinoma | High | Luminal B | Positive | Positive | 2 | Include |
| 49 | Invasive ductal carcinoma | High | Her 2 | Positive | Negative | 3 | Include |
| 50 | Invasive ductal carcinoma | High | Her 2 | Positive | Negative | 5 | Include |
| 51 | Invasive ductal carcinoma | High | Her 2 | Positive | Negative | 5 | Include |
| 52 | Invasive ductal carcinoma | High | Luminal B | Positive | Positive | 3 | Include |
| 53 | Invasive ductal carcinoma | High | Luminal B | Positive | Positive | 3 | Include |
| 54 | Invasive ductal carcinoma | High | Her 2 | Positive | Negative | 4 | Include |
| 55 | Invasive ductal carcinoma | High | TNBC | Negative | Negative | 5 | Include |
| 56 | Ductal carcinoma in situ | High | Luminal B | Positive | Positive | 5 | Include |
| 57 | Invasive ductal carcinoma | High | Luminal B | Positive | Positive | 5 | Include |
| 58 | Mixing invasive carcinoma | High | Luminal B | Positive | Positive | 3 | Include |
| 59 | Invasive ductal carcinoma | High | Luminal B | Negative | Positive | 2 | Include |
| 60 | Invasive ductal carcinoma | High | Luminal B | Positive | Positive | 3 | Include |
| 61 | Invasive ductal carcinoma | High | TNBC | Negative | Negative | 5 | Include |
| 62 | Invasive ductal carcinoma | High | Luminal B | Positive | Positive | 5 | Include |
| 63 | Invasive ductal carcinoma | High | Luminal B | Positive | Positive | 2 | Include |
| 64 | Invasive ductal carcinoma | High | TNBC | Negative | Negative | 5 | Include |
| 65 | Invasive ductal carcinoma | High | TNBC | Negative | Negative | 2 | Include |
| 66 | Invasive ductal carcinoma | High | Luminal B | Negative | Positive | 4 | Include |
| 67 | Invasive ductal carcinoma | High | Luminal B2 | Positive | Positive | 5 | Include |
| 68 | Invasive ductal carcinoma | High | Luminal B | Positive | Positive | 3 | Include |
| 69 | Invasive ductal carcinoma | Low | Luminal A | Positive | Positive | 1 | Include |
| 70 | Invasive ductal carcinoma | High | Her 2 | Positive | Negative | 3 | Include |
| 71 | Invasive ductal carcinoma | High | Luminal B | Positive | Positive | 3 | Include |
| 72 | Invasive ductal carcinoma | High | Her 2 | Positive | Negative | 5 | Include |
| 73 | Invasive ductal carcinoma | High | TNBC | Negative | Negative | 2 | Include |
| 74 | Invasive ductal carcinoma | High | Luminal B | Positive | Positive | 5 | Include |
| 75 | Invasive ductal carcinoma | High | Luminal B | Positive | Positive | 2 | Include |
| 76 | Invasive ductal carcinoma | High | Her 2 | Positive | Negative | 5 | Include |
| 77 | Invasive ductal carcinoma | High | Her 2 | Positive | Negative | 4 | Include |
| 78 | Invasive ductal carcinoma | High | Luminal B | Positive | Positive | 3 | Include |
| 79 | Invasive ductal carcinoma | High | Her 2 | Positive | Negative | 3 | Include |
| 80 | Invasive ductal carcinoma | High | Her 2 | Positive | Negative | 1 | Include |
| 81 | Invasive ductal carcinoma | High | Luminal B | Positive | Positive | 5 | Include |
| 82 | Invasive ductal carcinoma | High | Luminal B | Positive | Positive |  | Image incomplete |
| 83 | Invasive ductal carcinoma | High | Luminal B | Positive | Positive |  | Image incomplete |
| 84 | Invasive ductal carcinoma | High | Luminal A | Positive | Positive |  | Image incomplete |
| 85 | Invasive ductal carcinoma | High | Luminal B | Positive | Positive | 5 | Image incomplete |
| 86 | Invasive ductal carcinoma | High | TNBC | Negative | Negative |  | Image incomplete |
| 87 | Invasive ductal carcinoma | High | Luminal B | Positive | Positive |  | Image incomplete |
| 88 | Invasive ductal carcinoma | High | Luminal A | Positive | Positive |  | Image incomplete |
| 89 | Invasive ductal carcinoma | High | Her 2 | Positive | Negative |  | Image incomplete |
| 90 | Invasive ductal carcinoma | High | Luminal B | Positive | Positive |  | Image incomplete |
| 91 | Invasive ductal carcinoma | High | Luminal A | Positive | Positive |  | Image incomplete |
| 92 | Invasive ductal carcinoma |  |  |  |  |  | No MP grade results |
| 93 | Invasive ductal carcinoma |  | Luminal B |  |  |  | No MP grade results |
| 94 | Invasive ductal carcinoma |  |  |  |  |  | No MP grade results |
| 95 | Invasive lobular carcinoma |  |  |  |  |  | Imcomplete pathological results |
| 96 | Invasive ductal carcinoma |  |  |  |  |  | Imcomplete pathological results |
| 97 | Invasive ductal carcinoma | High | Luminal B | Positive | Positive |  | No pre-NAC images |
| 98 | Invasive ductal carcinoma |  | Luminal B |  |  |  | No pre-NAC images |
| 99 | Invasive ductal carcinoma | High | Luminal B | Positive | Positive |  | No pre-NAC images |
| 100 | Invasive ductal carcinoma | High | Luminal A | Positive | Positive | 1 | No pre-NAC images |
| 101 | Invasive ductal carcinoma |  |  |  |  |  | No pre-NAC images |
| 102 | Invasive ductal carcinoma | High | Luminal B | Positive | Positive |  | No pre-NAC images |
| 103 | Invasive ductal carcinoma |  |  |  |  |  | No pre-NAC images |
| 104 | Invasive ductal carcinoma | High | Luminal B | Positive | Positive | 5 | No pre-NAC images |
| 105 | Invasive ductal carcinoma | High | Luminal A | Positive | Positive |  | No pre-NAC images |
| 106 | Invasive ductal carcinoma |  | Luminal B |  |  |  | No pre-NAC images |
| 107 | Invasive ductal carcinoma | High | Luminal B | Positive | Positive |  | No pre-NAC images |
| 108 | Invasive ductal carcinoma | High | Luminal A | Positive | Positive |  | No pre-NAC images |
| 109 | Invasive ductal carcinoma |  |  |  |  |  | Incomplete clinical data |
| 110 | Invasive ductal carcinoma | High | Luminal B | Positive | Positive |  | Incomplete clinical data |
| 111 | Invasive ductal carcinoma | High | Her 2 | Positive | Negative | 2 | Incomplete clinical data |
| 112 | Invasive ductal carcinoma |  |  |  |  |  | Incomplete clinical data |
| 113 | Invasive ductal carcinoma |  | Luminal B |  |  |  | Incomplete clinical data |
| 114 | Invasive ductal carcinoma | High | Luminal B | Positive | Positive |  | Incomplete clinical data |
| 115 | Invasive ductal carcinoma | High | Luminal B | Positive | Positive | 2 | Incomplete clinical data |
| 116 | Invasive ductal carcinoma |  |  |  |  |  | Incomplete clinical data |
| 117 | Invasive ductal carcinoma |  | Her 2 |  |  |  | Image incomplete |
| 118 | Invasive ductal carcinoma |  |  |  |  |  | Image incomplete |
| 119 | Invasive ductal carcinoma |  | Luminal B |  |  |  | Image incomplete |
| 120 | Invasive ductal carcinoma | High | Luminal B | Positive | Positive |  | Image incomplete |
| 121 | Invasive ductal carcinoma | High | Her 2 | Positive | Negative | 5 | Image incomplete |

Patients data. ER Estrogen receptor; PR Progesterone receptor; HER-2, human epidermal growth factor receptor; TNBC, triple negative breast cancer; MP, Miller-Payne Grading system.

| **Mid-NAC** | **AUC** | **P-value** |  | **SEN** |  | **SPE** |  | **Cutoff** |
| --- | --- | --- | --- | --- | --- | --- | --- | --- |
| Diameter (mm) | 0.699(0.558, 0.841) | 0.012 |  | 0.5 |  | 0.875 |  | 31.5 |
| Volume (mm3) | 0.751(0.616, 0.886) | 0.002 |  | 0.6 |  | 0.917 |  | 6561.1 |
| Segmented volume (mm3) | 0.729(0.591, 0.868) | 0.004 |  | 0.767 |  | 0.708 |  | 1520.4 |
| Segmented volume reduction (%) | 0.677(0.524, 0.830) | 0.026 |  | 0.5 |  | 0.903 |  | 0.915 |
| Segmented surface area (mm2) | 0.697(0.554, 0.841) | 0.013 |  | 0.633 |  | 0.792 |  | 4309.2 |
| Segmented surface area reduction (%) | 0.617(0.463, 0.771) | 0.140 |  | 0.542 |  | 0.742 |  | 0.805 |
| Washout rate (%) | 0.649(0.503, 0.796) | 0.06 |  | 0.583 |  | 0.71 |  | 0.217 |
| Maximum enhancement ratio (%) | 0.663(0.516, 0.809) | 0.04 |  | 0.613 |  | 0.75 |  | 1.265 |
| Washin rate (%) | 0.667(0.523, 0.812) | 0.035 |  | 0.774 |  | 0.542 |  | 0.075 |
| One-min enhancement (HU) | 0.593(0.441, 0.746) | 0.238 |  | 0.645 |  | 0.625 |  | 21 |
| Two-min enhancement (HU) | 0.759(0.630, 0.888) | 0.001 |  | 0.645 |  | 0.792 |  | 42 |
| Three-min enhancement (HU) | 0.644(0.491,00.797) | 0.069 |  | 0.774 |  | 0.542 |  | 20.5 |
|  |  |  |  |  |  |  |  |  |

|  |  |  |  |  |  |  |  |  |
| --- | --- | --- | --- | --- | --- | --- | --- | --- |

|  |  |  |  |  |  |  |  |  |
| --- | --- | --- | --- | --- | --- | --- | --- | --- |
|  |  |  |  |  |  |  |  |  |
|  |  |  |  |  |  |  |  |  |
|  |  |  |  |  |  |  |  |  |
|  |  |  |  |  |  |  |  |  |
|  |  |  |  |  |  |  |  |  |
|  |  |  |  |  |  |  |  |  |
|  |  |  |  |  |  |  |  |  |
|  |  |  |  |  |  |  |  |  |

Table One. Prediction performance of pCR with CBBCT parameters in mid-NAC. HU, ounsfield unit. AUC, The area under the

receiver operator characteristic curve. SEN, sensitivity. SPE, specificity. CBBCT, cone beam breast computed tomography. NAC,

neoadjuvant chemotherapy. pCR, pathologic complete response.

| **Late-NAC** | **AUC** | **P-value** |  | **SEN** |  | **SPE** |  | **Cutoff** |
| --- | --- | --- | --- | --- | --- | --- | --- | --- |
| Diameter (mm) | 0.696(0.567, 0.825) | 0.007 |  | 0.667 |  | 0.69 |  | 16.5 |
| Volume (mm3) | 0.689(0.559, 0.818) | 0.009 |  | 0.806 |  | 0.552 |  | 539.3 |
| Segmented volume (mm3) | 0.791(0.679,0.904) | <0.001 |  | 0.75 |  | 0.862 |  | 970.9 |
| Segmented volume reduction (%) | 0.761(0.645, 0.878) | <0.001 |  | 0.611 |  | 0.862 |  | 0.865 |
| Segmented surface area (mm2) | 0.766(0.652,0.881) | <0.001 |  | 0.667 |  | 0.828 |  | 1705.2 |
| Segmented surface area reduction (%) | 0.744(0.626, 0.863) | 0.001 |  | 0.528 |  | 0.862 |  | 0.875 |
| Washout rate (%) | 0.536(0.394, 0.679) | 0.616 |  | 0.379 |  | 0.778 |  | 0.314 |
| Maximum enhancement ratio (%) | 0.837(0.735, 0.938) | <0.001 |  | 0.966 |  | 0.667 |  | 1.25 |
| Washin rate (%) | 0.762( 0.647, 0.878) | <0.001 |  | 0.966 |  | 0.528 |  | 7.5 |
| One-min enhancement (HU) | 0.717(0.593, 0.841) | 0.003 |  | 0.645 |  | 0.625 |  | 21 |
| Two-min enhancement (HU) | 0.806(0.702, 0.910) | <0.001 |  | 0.645 |  | 0.792 |  | 42 |
| Three-min enhancement (HU) | 0.830(0.728, 0.933) | <0.001 |  | 0.774 |  | 0.458 |  | 20.5 |

Table Two. Predictive performance of pCR with CBBCT parameters in late-NAC. HU, hounsfield unit. AUC, The area under the receiver operator characteristic curve. SEN, sensitivity. SPE, specificity. CBBCT, cone beam breast computed tomography. NAC, neoadjuvant chemotherapy. pCR, pathologic complete response.

|  | **OR** | **SE** | **P-value** |
| --- | --- | --- | --- |
| (Reference) | 1 |  |  |
| Hormal status | 0.245(0.079,0.762) | 0.578 | 0.015 |
| Two-min enhancement (HU) | 0.989(0.978, 0.999) | 0.005 | 0.03 |
| Three-min enhancement (HU) | 1.006(0.988, 1.024) | 0.009 | 0.527 |
| Washin rate | 0.015(0,1.175) | 2.23 | 0.059 |
| Maximum enhancement ratio | 0.06(0.004,0.966) | 1.422 | 0.047 |
| Diameter | 0.954(0.917, 0.992) | 0.02 | 0.019 |
| Segmented volume | 0.987(0.952,1.023) | 0.018 | 0.462 |
| Segmented surface area | 0.997(0.97,1.025) | 0.014 | 0.835 |
| Segmented volume reduction | 0.971(0.934, 1.009) | 0.020 | 0.138 |
| Segmented surface area reduction | 0.982(0.948, 1.016) | 0.017 | 0.288 |

Table Three. Univariable analysis of CBBCT parameters in mid-NAC. HU, hounsfield unit. OR, odds ratio. SE, standard error. CBBCT, cone beam breast computed tomography. NAC, neoadjuvant chemotherapy.

Table Three. Univariable analysis of CBBCT parameters in mid-NAC. HU, hounsfield unit. OR, odds ratio. SE, standard error. CBBCT, cone beam breast computed tomography. NAC, neoadjuvant chemotherapy.
